# Evaluation of the children with acute acetaminophen overdose and intravenous N-acetylcysteine treatment

**DOI:** 10.12669/pjms.343.14937

**Published:** 2018

**Authors:** Yakup Yesil, Abdurrahman Avar Ozdemir

**Affiliations:** 1 Yakup Yesil, MD. Department of Pediatrics, Kanuni Sultan Suleyman Training and Research Hospital, Medical Sciences University, Turkey.; 2 Abdurrahman Avar Ozdemir Department of Pediatrics, Medicine Hospital, Biruni University, Turkey

**Keywords:** Acetaminophen intoxication, N- acetylcysteine, Children

## Abstract

**Objective:**

To evaluate the demographic and clinical features associated with acetaminophen overdose and to identify the clinical use of IV (intravenous) N- Acetylcysteine (NAC) treatment in children.

**Methods:**

This prospective study was conducted in Kanuni Sultan Suleyman Training and Research Hospital between August 2016 and August 2017. A total of 59 patients with overdose acetaminophen ingestion were included in this study. The toxic dose for acute acetaminophen intake was defined as greater than 150 mg/kg. Rumack-Matthew nomogram was used to evaluate the risk of acute intoxication and to determine the decision of using antidote.

**Results:**

The mean age of the patients was 8.5±6.4 y and 34 of them (58%) were female. The mean time from ingestion to admission was 4.3±4.7 h. The mean ingested acetaminophen dose was 142.1±80 mg/kg. Twenty four patients (41%) received NAC and there were significant differences in terms of acetaminophen dose, creatinine and INR between antidote and decontamination therapy groups at admission time (p= 0.00, p= 0.03, p= 0.02, respectively). The complication due to antidote therapy was observed in only 1 patient.

**Conclusions:**

This study confirms that the side effects due to IV NAC therapy are uncommon and it is generally well tolerated in children.

## INTRODUCTION

Acetaminophen is commonly used in children as an analgesic and antipyretic. Although its’ safety was well established, it is one of the common cause of drug poisoning. Repeated supra-therapeutic dosing and intentional or unintentional overdose usage may result in hepatic failure.^1^

Acetaminophen is metabolized by glucuronidation and sulfation in liver. However, approximately 5-10% of the drug is metabolized by Cytochrome P450 (CYP450) to a toxic metabolite, N-acetyl-p-benzoquinoneimine (NAPQI). When acetaminophen is used at the recommended doses, NAPQI is eliminated by conjugation with glutathione. If it is used overdose, glutathione stores are depleted and the clearance of NAPQI is reduced. As a result, this toxic metabolite accumulates and causes hepatocellular injury.^2^ Management of acetaminophen overdose includes; gastric lavage, using of activated charcoal and N-Acetylcysteine (NAC) as an antidote.^1^-^3^

The aim of this study was to evaluate demographic and clinical features associated with acetaminophen overdose as well as to identify the treatment applied in our Pediatric Emergency Unit.

## METHODS

This prospective study was conducted in Kanuni Sultan Suleyman Training and Research Hospital between August 2016 and August 2017. The study protocol was approved by the local ethics committee and informed consent was obtained for all children from their parents. A total of 59 patients who came to Pediatric Emergency Unit with overdose acetaminophen ingestion were included in this study. The exclusion criteria were refusal of treatment or informed consent. Age, gender, weight, amount of the ingested acetaminophen, co-ingested substances, time elapsed from ingestion, method of gastrointestinal decontamination, and antidote usage were recorded.

Amount of ingested acetaminophen was reported as milligrams per kilogram and toxic dose for acetaminophen intake was defined as greater than 150 mg/kg.^2^ Rumack-Matthew nomogram for acute acetaminophen intake was used to evaluate the risk of acute intoxication and to determine the decision of using antidote (NAC).^4^ Decontamination (lavage and activated charcoal) and Intravenous (IV) NAC therapy was administered to patients who were exposed to toxic dose. Laboratory data included serum acetaminophen level, whole blood count, aspartate Aminotransferase (AST) level, alanine aminotransferase (ALT) level, serum urea level, creatinine level, Prothrombin (PT) time, activated Partial Thromboplastin Time (aPTT) and international normalized ratio (INR). Whole blood count was measured on the Cobas 6000 CE (Roche, Germany) and XN-10 (Sysmex, Japan) was used to measure ALT, AST, urea and creatinine. Blood samples for acetaminophen levels were measured in laboratory of Bakırköy Mental Health and Neurology Training and Research Hospital by a multipoint homogeneous immunoassay on the Advia 1800 (Siemens, Japan).

SPSS Statistics 20 was used for data analysis. Descriptive statistics were given as mean, standard deviation, minimum, maximum. The significance between groups were evaluated with χ^2^-test for qualitative data and Mann-Whitney U test for quantitative data. Correlations between quantitative data were analyzed by Spearman’s correlation test.

## RESULTS

During the study period, 137 patients who presented for analgesics/antipyretics overdose in our hospital and we found that 59 (43%) of them ingested the drugs containing acetaminophen. The mean age of the patients was 8.5±6.4 y (min-max; 1-17) and 34 of them (58%) were female. The patients were divided into 2 groups according to age; 34 of them (52%) were in 0-8, 25 (48%) of them were in 8-17 years age group. There was male dominance (30; 88%) in 0-8 age group, whereas there was female dominance (21; 84%) in 8-17 age group. The mean weight of the patients was found as 32.9±22 kg (min-max; 9-80) (Table-I).

**Table-I:** Demographics and clinical characteristics of patients.

	n (%)	Acetaminophen dose (mg/kg) (Mean±SD)	p
Age (y) (Mean±SD)	8.5±6.4	142±80	^1^0.06
0-8	34 (58%)	163 ± 89	
≥9-16	25 (42%)	112.6 ± 53	
Gender n (%)			^1^0.08
Male	34 (58%)	164±84	
Female	25 (42%)	125±73	
Weight (kg) (Mean±SD)	32.9±22.9		^1^0.05
≤25	32 (54%)	165±90	
>25	27 (46%)	112±52	
Ingested Substance			^1^0.008
Acetaminophen only	29 (49%)	174± 89	
Acetaminophen and additional drug	30 (51%)	109±53	
Time from ingestion to admission (Mean±SD)	4.3±4.7		^1^0,9
≤8 h	49 (83%)	142±81	
>8 h	19 (17%)	138±75	

1 Mann-Whitney U Test.

The mean time from ingestion of the drug to admission was found as 4.3±4.7 h (min-max; 0.5-20); 50 (85%) of patients came in 8 h and all of them came to hospital in 24 h. When the admission hours were analyzed; 25 of them admitted to hospital between 18:00-24:00, 17 of them between 12:00-18:00 hours, 9 of them between 00:00-06:00 hours and 8 of them between 06:00-12:00 o’clock. Twenty nine of them (49%) ingested only acetaminophen but others (30, 51%) ingested more than one drug. The mean ingested acetaminophen dose was found as 142.1±80 mg/kg (min-max; 24-300). There were no significant differences between groups (age, gender, weight, admission mean time) in terms of ingested acetaminophen dose. However, there was significantly difference between ingested only acetaminophen and additional drug groups. (p= 0.008) (Table-I).

Acetaminophen concentrations were measured from the 22 patients (37%) who ingested more than 150 mg/kg. Mean serum acetaminophen level was found as 14.3±23 µg/mL (min-max; 0.2-107). When the laboratory parameters were evaluated, the liver enzymes’ elevation was found in 1 patient and INR elevation was found in another patient. Also, we analyzed correlations between ingested acetaminophen dose and time from ingestion to admission, serum acetaminophen level, ALT, AST, urea, creatinine, INR. But there were no significant correlation (r: 0.00; p= 0.98, r: 0.11; p= 0.6, r: 0.15; p= 0.95, r: 0.076; p= 0.74, r: 0.32; p= 0.12, r: 0.13; p= 0.59, r: 0.18; p= 0.53, r:0.17, respectively).

Twenty four patients (41%) received IV NAC therapy as well as decontamination. Patients receiving NAC therapy were compared with those receiving only decontamination therapy (35; 59%). There were significant differences in acetaminophen dose, creatinine and INR between groups at admission time (p= 0.00, p= 0.03, p= 0.02, respectively) (Table-II). However, there were no significant differences in terms of creatinine and INR between groups at discharged time (p= 0.9, p= 0.06, respectively). We observed urticarial reaction as a complication due to antidote therapy in only 1 patient.

**Table-II:** Clinical and demographic characteristics of patients according to treatment methods.

	Decontamination therapy (Mean±SD)	Decontamination and NAC therapy (Mean±SD)	Total	p
Age (y)	7.2±5.9	10±6.7	8.5±6.4	^1^0.07
Gender n (%) Male Female	15 (43%) 20 (57%)	10 (42%) 14 (58%)	25 (42%) 34 (58%)	^2^0.85
Weight (kg)	27.5±20	39.2±25	35.1±23	^1^0.07
Substance ingested n (%) Acetaminophen only Additional drug	15 (43%) 20 (57%)	14 (58%) 10 (42%)	29 (49%) 30 (51%)	^2^0.4
Time from ingestion to admission (h)	4.3±4.7	4.6±4.9	4.4±4.8	^1^0.8
Acetaminophen dose (mg/kg)	106±53	191±85	142±80	^1^0.00
Acetaminophen level (µg/mL)	13±11	14.7±26	14.3±23	^1^0.2
Hb (gr/dL)	12.5±0,9	12.4±1.7	12.5±1.4	^1^0.8
ALT (UI/L)	17.5±8.7	21±25.8	19±18	^1^0.9
AST (UI/L)	23.5±9.3	21.5±9.7	22.6±9.4	^1^0.3
Urea (mg/dL)	22.9±8.5	24.7±7.4	23.7±8	^1^0.2
Creatinine (mg/dL)	0.43±0.16	0.55±0.2	0.49±0.2	^1^0.03
INR	1.0±0.1	1.1±0.09	1.1±0.1	^1^0.02
Total n (%)	35 (59%)	24 (41%)	58 (100%)	

1 Mann-Whitney U test,

2 Chi-Square Test of Independence.

Twenty two (37%) of all patients ingested the medicines due to suicide attempt. The mean age of these were found as 15.8±1 y (range 12-17) and 17 of them (77%) were female. The number of patients received NAC therapy were found as 12 (55%). Seventeen (77%) of the patients ingested multi drugs for suicide. All of patients who attempt suicide were referred to psychiatric service for consultation. The total number of the ingested additional drugs together with acetaminophen were 47; 14 (30%) of them were anti-gripal, 9 (19%) of them were gastrointestinal drugs, 7 (15%) were nonsteroidal anti-inflammatory (NSAI), 6 (13%) were antibiotic, 4 (9%) were antidepressant, 2 (4%) were steroid, and 5 (10%) were others.

When the signs and symptoms of the patients were evaluated, nausea and abdominal pain were determined in 38 (64%) of the patients. Other common symptoms were vomiting, headache and fatigue. One patient with neurologic disorder had convulsion. However, 21 (36%) of them were asymptomatic. Total number of patients admitted to inpatient service was 18 and 2 of these admitted to pediatric intensive care unit due to additional drugs. In follow up, clinical course and laboratory parameters of all patients were resolved. None of patients died.

## DISCUSSION

Analgesics are the most common substance that causes poisoning in all ages. When the child age group is evaluated, analgesic intoxication is third after the cosmetics and cleaning substances for younger than 5 years old children according to the 2014 annual report of the American Association of Poison Control Centers. 5 Similarly, acetaminophen was reported as the drug most frequently used in self-poisoning in UK and Spain.^6^,^7^ Unfortunately, national data on intoxications in Turkey is limited. The number of deaths in 2016 due to intoxications were 855; the drugs were the most frequent cause for intoxications and acetaminophen was leading agent according to the report of National Poison Consultation Center (UZEM) and regional studies.^8^-^14^

In our study, 684 patients admitted to our emergency unit due to intoxication with drugs. Similar to previous studies, we found that analgesics/antipyretics were the most frequently (20%) ingested drugs and acetaminophen was the first (43%) cause among them. The mean age of the patients was 8.5±6.4 y and it was higher than previous studies.^10^,^12^,^13^ This result may be associated with distribution of patients’ ages. Because, there were no patients between 7.5-13 years age in our study. The number of patients in the 0-8 age group constituted the majority and there was a male predominance. However, there was a female predominance in 8-17 age group and the most frequent reason for intoxication was suicide attempt. Similar data were obtained from other studies in our country.^11^-^14^ These results may be related to emotional pressure and conflicts that girls experience during adolescence. We found that the rate of multidrug ingestion was 51%. When the multidrug ingestion was evaluated, the anti-gripal drugs accounted for 30% of them. In previous studies, the ingestion of multidrug was reported as 5-19%.^12^,^13^,^15^,^16^ However, those studies included all substance exposures.

The mean time from ingestion to hospital admission was found as 4.3 h. and the ingested acetaminophen dose was significantly higher in patients who ingested only acetaminophen. As expected, the ingestion of more than one agent reduces the proportion of acetaminophen in the total dose. No correlations were determined between laboratory parameters and ingested dose. This result might be related with all of patients admitted to hospital due to acute intoxication, shortened admission time and early intervention.

Early studies showed that NAC is an effective therapy for acetaminophen toxicity and it initiated within first 8 hours decreases a 10% incidence of hepatotoxicity and usually liver failure does not develop. It can be given oral or IV route and adverse reactions were uncommon.^17^,^2^,^3^ Although, there is no consensus on the duration of treatment, if it is initiated within 8 h after poisoning, there is no difference between 21 h and the longer treatment.^3^,^18^ In our hospital, we used 21 h protocol for I.V NAC treatment. In our study, 41% of the patients received IV NAC as well as decontamination therapy. Unfortunately, the results of serum acetaminophen level were obtained after 1 day because the blood samples sent to another hospital laboratory. The previous studies in children showed that the risk of hepatotoxicity is lower than adult patient and late presentation (>24 h after ingestion) or delayed therapy are an important risk factors.^19^-^21^ Therefore, we decided to initiate NAC therapy based on the ingested acetaminophen dose and clinical opinion.

The studies in Turkey have been conducted in the adult age group and are limited in number. Duran et al. reported that nausea or vomiting were determined in 56.6% of the patients and the mean time of therapy initiation was 5.8 h. Hepatotoxicity developed in 1 patient and complication due to NAC therapy (urticarial reaction) was observed in 1 patient.^22^ Karaman et al. reported that hepatotoxicity, nephrotoxicity or death were not observed in any patients.^23^ In our study, we found that the most common findings were nausea, vomiting and abdominal pain. The mean time from ingestion to initiation of NAC therapy was found as 4.6±4.9 h and none of the patients developed hepatotoxicity. This short admission period may also explain that 36% of the patients were asymptomatic. Similarly, urticarial reaction was observed in one patient. When the patients who received NAC and only decontamination therapy were compared, as expected we found the ingested acetaminophen dose and serum acetaminophen level were higher in patients who received antidote. Some previous studies reported that INR values were higher in patients with acetaminophen poisoning without liver damage but these values reduced after NAC therapy.^23^-^25^ We found that creatinine and INR values were slightly high in patients received NAC therapy at admission time. However, there were no differences in terms of creatinine and INR between groups at discharge.

There are some limitations that should be mentioned in our study. The results of serum acetaminophen level were obtained after 1 day and this delay might have affected treatment choice.

As a result, this study confirm that the incidence of side effects due to IV NAC therapy are uncommon and it is generally well tolerated in children. Additionally, we found that anti-gripal drugs are in the first place in multi-drug ingestion. It is necessary to avoid the prescription of such drugs containing more than one agent because dosing errors can be made easily.

### Authors’ Contribution

**YY:** Designed and did statistical analysis, writing & editing of manuscript.

**AAO:** Did data collection and manuscript writing.
